# Effect of trait’s expression level on single-step genomic evaluation of resistance to Dothistroma needle blight

**DOI:** 10.1186/s12870-020-02403-6

**Published:** 2020-05-11

**Authors:** Jaroslav Klápště, Heidi S. Dungey, Natalie J. Graham, Emily J. Telfer

**Affiliations:** Scion (New Zealand Forest Research Institute Ltd.), 49 Sala Street, Rotorua, 3010 New Zealand

**Keywords:** Single-step genomic evaluation, Exome capture, Needle disease resistance, *Pinus radiata*, Dothistroma needle blight

## Abstract

**Background:**

Many conifer breeding programs are paying increasing attention to breeding for resistance to needle disease due to the increasing importance of climate change. Phenotyping of traits related to resistance has many biological and temporal constraints that can often confound the ability to achieve reliable phenotypes and consequently, reliable genetic progress. The development of next generation sequencing platforms has also enabled implementation of genomic approaches in species lacking robust reference genomes. Genomic selection is, therefore, a promising strategy to overcome the constraints of needle disease phenotyping.

**Results:**

We found high accuracy in the prediction of genomic breeding values in the disease-related traits that were well characterized, reaching 0.975 for genotyped individuals and 0.587 for non-genotyped individuals. This compared well with pedigree-based accuracies of up to 0.746. Surprisingly, poorly phenotyped disease traits also showed very high accuracy in terms of correlation of predicted genomic breeding values with pedigree-based counterparts. However, this was likely caused by the fact that both were clustered around the population mean, while deviations from the population mean caused by genetic effects did not appear to be well described. Caution should therefore be taken with the interpretation of results in poorly phenotyped traits.

**Conclusions:**

Implementation of genomic selection in this test population of *Pinus radiata* resulted in a relatively high prediction accuracy of needle loss due to *Dothistroma septosporum* compared with a pedigree-based approach. Using genomics to avoid biological/temporal constraints where phenotyping is reliable appears promising. Unsurprisingly, reliable phenotyping, resulting in good heritability estimates, is a fundamental requirement for the development of a reliable prediction model. Furthermore, our results are also specific to the single pathogen mating-type that is present in New Zealand, and may change with future incursion of other pathogen varieties. There is no doubt, however, that once a robust genomic prediction model is built, it will be invaluable to not only select for host tolerance, but for other economically important traits simultaneously. This tool will thus future-proof our forests by mitigating the risk of disease outbreaks induced by future changes in climate.

## Background

Dothistroma needle blight (DNB), also known as red band needle blight, is one of the most important needle diseases that affect conifer species (pines) across the world [1–4]. Frequency and severity of reports have been on the rise since the early 1990s, including new locations and host species [3]. The severity of DNB has put pressure on the productivity of pine plantations and led to the abandonment of further deployment of a number of pine species across the world [3].

Dothistroma needle blight is caused by the pathogen *Dothistroma septospora* (Dorog.) Morelet and characterized by 1–3 mm wide brick-red bands around the needles, caused by the release of a mycotoxin (dothistromin) [5]. Infection of *Pinus radiata* D. Don. by this pathogen is dependant on climate [6]. High levels of infection follow periods combining both warm temperatures and needles remaining wet [7, 8]. Older needles found closer to the main stem are more affected than younger needles and infection can be spread from affected needles to other trees via wind. The disease causes large losses in growth, the loss increasing in proportion with the degree of affected crown, with van der Pas [9] reporting 1% loss in productivity for each 1% increase in disease level and a significant reduction in stand growth once defoliation exceeds 25 percent [9].

Previous studies have found moderate genetic control in resistance of *P. radiata* to DNB [10–12] and breeding for resistance in the *P. radiata* breeding program is recommended for high-risk areas [11]. Ivković et al. [4] found a negative genetic correlation of -0.39 between resistance to DNB at an early age and productivity at a later age, translating to high economic losses. This is despite *P. radiata* naturally developing resistance to Dothistroma at around age 15 [13, 14] possibly due to structural changes in wax surface topography and stomatal organization [15]. Therefore, reliable phenotyping of traits related to Dothistroma resistance at early ages remains important. Similar to other needle diseases [16], phenotyping during the optimum spread and intensity of disease is vital for any genetics study, and the ability to achieve this remains confounded by annual variability in the weather conditions and the narrow window of time available for quality phenotyping.

Genomic selection (GS) has been proposed as a tool to predict phenotypes based on genetic markers obtained from whole genome sequencing [17]. The method is based on the capture of genealogy (both historical and contemporary [18]), co-segregation and linkage disequilibrium between markers and quantitative trait loci (QTL) [19]. The development of next-generation sequencing technologies such as genotyping by sequencing [20] or exome capture [21] has allowed the development of genomic resources and the implementation of genomic selection approaches in species without reference genomes, a common limitation in conifers [22–29]. The efficiency of genomic selection depends on the heritability of a trait, the size of the population used for training the prediction model and genetic distance between the training population and material being predicted [30]. Forest tree progeny tests are typically large populations containing thousands of individuals, which has prevented full-scale genotyping [25]. Therefore, the combination of phenotype, pedigree and genomic information through a single-step evaluation approach [31] is the preferred strategy in forest tree breeding [32–34].

The aim of this study was to present a proof-of-concept for the implementation of genomic selection for traits where biology and the environment constrains genetic progress in typical tree breeding approaches. We use the example of phenotyping expression of a needle disease (*Dothistroma septosporum*) on *Pinus radiata* to illustrate this approach.

## Results

### Genetic parameters and model fit

**Fig. 1:**
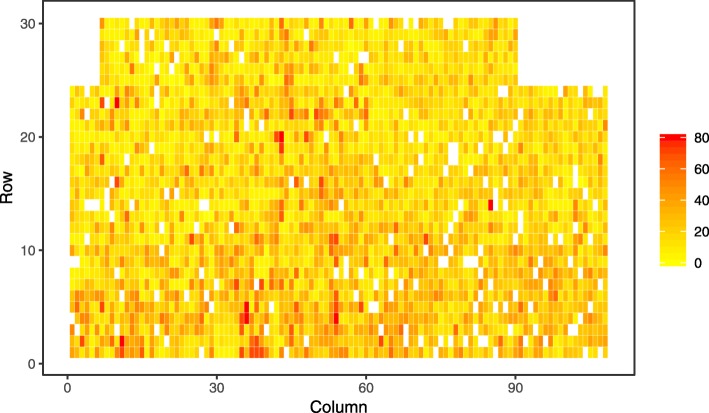
Spatial distribution of phenotypes at Kaingaroa. Spatial distribution of phenotypes measured as percentage of crown affected by Dothistroma needle blight at Kaingaroa site at age 2

**Fig. 2:**
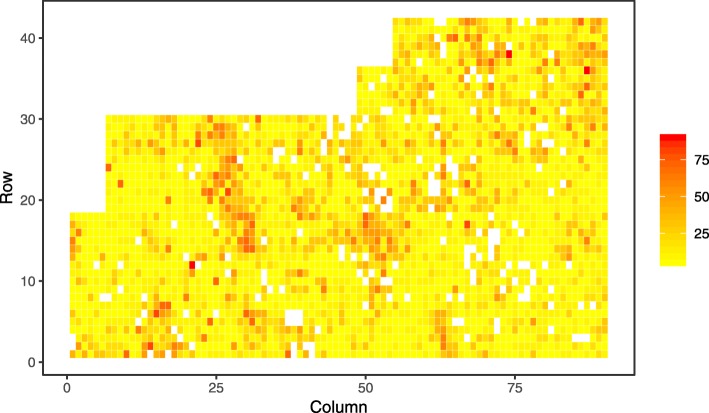
Spatial distribution of phenotypes at Kinleith 1. Spatial distribution of phenotypes measured as percentage of crown affected by Dothistroma needle blight at Kinleith 1 site at age 2

**Fig. 3:**
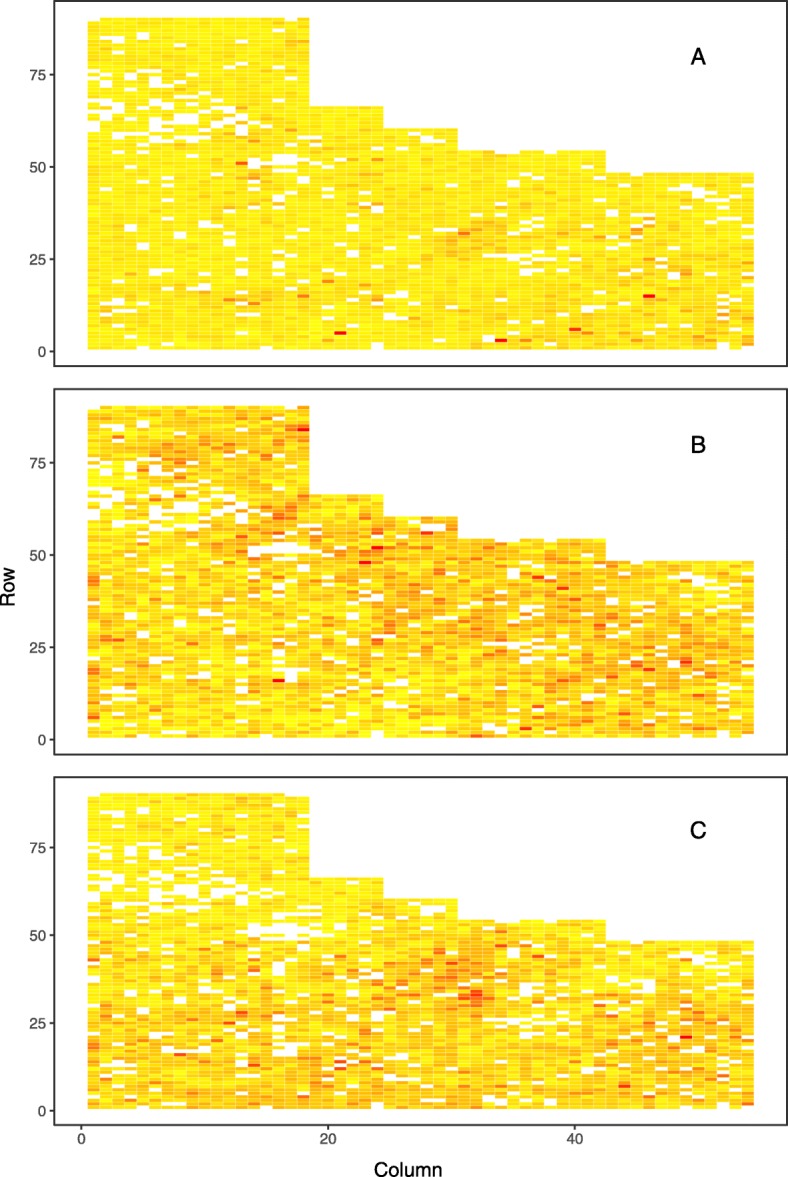
Spatial distribution of phenotypes at Kinleith 2. Spatial distribution of phenotypes measured as percentage of crown affected by Dothistroma needle blight at Kinleith 2 site at age 2 (plot **A**), age 3 (plot **B**) and age 4 (plot **C**)

**Fig. 4:**
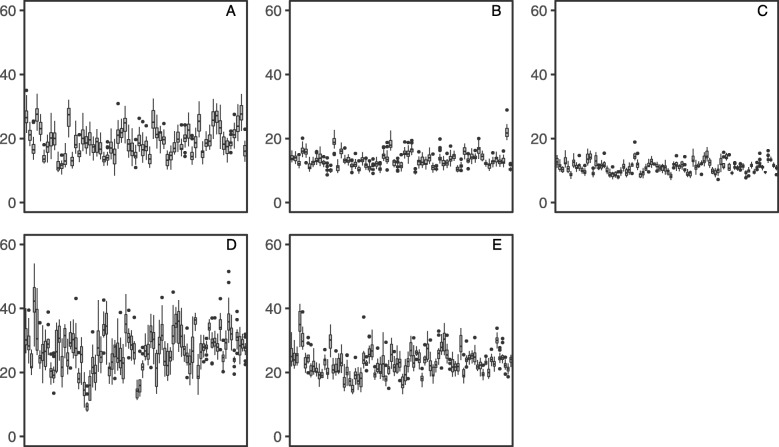
Distribution of pedigree-based breeding values. Family-wise distribution of pedigree-based breeding values estimated at each site: Kaingaroa at age 2 (plot **A**), Kinleith 1 at age 2 (plot **B**), Kinleith 2 at age 2 (plot **C**) Kinleith 2 at age 3 (plot **D**) and Kinleith at age 4 (plot E)

A visual exploration of spatial patterns in the level of phenotypic expression of disease found the lowest level at an early age (two years after planting) across all investigated sites with exception of Kaingaroa (Figs. 1, 2 and 3). This exception can be explained by higher intensity of attack at Kaingaroa compared to Kinleith sites. These findings were reflected in the relatively low heritability at Kinleith sites at age 2, from 0.188 to 0.219, compared with the moderate heritability at later ages, from 0.309 to 0.429, as well as 0.321 at Kaingaroa at age of two (Table 1). The non-additive genetic component reached a moderate level, between 13% (Kinleith 1 and Kinleith 2) and 37% (Kaingaroa) of the additive genetic variance. This pattern corresponded to the trends apparent in the broad-sense heritability, ranging from 0.212 (Kinleith 1 - age 2) to 0.536 (Kinleith 2 - age 3). Exploration of the breeding values estimated on the basis of the pedigree-based analysis found strong shrinkage of breeding values toward the population mean where heritability was low (Kinleith 1 - age 2) and a corresponding wider dispersion in populations where heritability was higher (Kinleith 2 - age 3) (Fig. 4).

**Table 1:** Variance components, narrow-sense and broad-sense heritability, their standard errors in parenthesis, column and row autocorrelations and model fit in terms of Akaike’s Information Criterion (AIC) estimated for Dothistroma needle blight resistance at each site and age

	**Kaingaroa**	**Kinleith 1**	**Kinleith 2**	**Kinleith 2**	**Kinleith 2**
**Parameter**	**Age 2**	**Age 2**	**Age 2**	**Age 3**	**Age 4**
**Add.Gen.Var.**	0.678 (0.182)	0.317 (0.096)	0.165 (0.045)	81.20 (19.12)	36.13 (9.188)
**Non-add.Gen.Var.**	0.399 (0.108)	0.041 (0.066)	0.022 (0.028)	20.18 (10.83)	6.984 (5.428)
**Block Var.**	0.032 (0.020)	0.122 (0.051)	0.018 (0.012)	0.000 (0.000)	2.887 (1.545)
**Residual Var.**	1.036 (0.043)	1.327 (0.068)	0.566 (0.020)	88.01 (3.259)	78.12 (2.670)
**AR1 col**	0.913	0.659	0.796	0.838	0.893
**AR1 row**	0.824	0.738	0.852	0.872	0.918
***h***^**2**^	0.321 (0.073)	0.188 (0.052)	0.219 (0.053)	0.429 (0.081)	0.298 (0.066)
***H***^**2**^	0.509 (0.027)	0.212 (0.033)	0.249 (0.027)	0.536 (0.028)	0.356 (0.030)
**AIC**	4644	4961	2451	19474	18097
***h***^2^*	0.316 (0.072)	0.161 (0.045)	0.211 (0.026)	0.370 (0.074)	0.270 (0.061)
AIC*	4737	5108	2562	19727	18327
**Phenotypic mean**	21.1	15.5	12.2	27.4	23.4
**Phenotypic st. dev.**	14.56	14.64	7.78	15.49	13.14

Two models were investigated: using experimental design terms (parameters with the star) and spatial analysis

Investigation of the optimal weighting of pedigree and genomic information through model fit in terms of DIC criterion found that the best model mostly differed to the default (0.05 weight put on pedigree-based relationship matrix). There was also a tendency to place a higher weight on the documented pedigree with decreasing heritability, resulting in an increasing accumulation of breeding values around the population mean. There was only one exception, where the best scenario was the default weighting, and this was for the population with the lowest heritability (Kinleith 1 - age 2). In general, the optimal weighting scenario defined by the lowest DIC criterion was reached at a weight of 0.4 - 0.6 for the documented pedigree in populations with low to moderate heritability, and for populations with a higher heritability, the best weighting was 0.2 (Table 2).

**Table 2:** Deviance Information criterion (DIC) scores obtained for each tested scenario using genotypic values, bold DIC score represents scenario with best model fit (HBLUP1) (lower values represent better model fit)

**Weight**	**Kaingaroa**	**Kinleith 1**	**Kinleith 2**	**Kinleith 2**	**Kinleith 2**
**on pedigree**	**Age 2**	**Age 2**	**Age 2**	**Age 3**	**Age 4**
**0.05**	2886	225	-170	6270	5313
**0.10**	2884	223	-180	6263	5312
**0.20**	2877	220	-192	6262	5298
**0.30**	2877	217	-185	6272	5307
**0.40**	2867	218	-192	6278	5298
**0.50**	2871	215	-201	6285	5296
**0.60**	2867	215	-183	6305	5303
**0.70**	2875	215	-180	6329	5317
**0.80**	2883	221	-166	6465	5340
**0.90**	2902	222	-149	6365	5359
**1.00**	2917	228	-123	6443	5388

### Predictive ability

The ability to predict appeared to reflect a trend in heritability across all populations and was at its lowest in the population with the lowest heritability. The predictive ability at Kinleith 1 - age 2 (the population with the lowest heritability) was as low as 0.260 using ABLUP and 0.259 using HBLUP. Higher predictive ability was possible for genotyped individuals (0.285) compared with non-genotyped individuals. The ability to predict phenotypes gradually increased with the increasing heritability of the studied population and reached 0.454 using ABLUP, 0.488 using HBLUP and 0.485 using HBLUP1 (the optimal scenario) in a population with the highest heritability. The lower predictive ability that was found in the optimal scenario based on model fit (DIC) appeared to be caused by a decreased predictive ability in non-genotyped individuals, with a corresponding increase in genotyped individuals compared with the default HBLUP scenario.

The predictive ability of GS prediction models between environments and ages showed the clear impact of phenotyping precision (in terms of each traits’ heritability). The lowest predictive ability was at Kinleith 1 - age 2, with values from 0.174 to 0.199 for non-genotyped individuals and from 0.268 to 0.298 for genotyped individuals. As expected, the highest was at Kinleith 2 between ages 3 and 4 (0.292) for non-genotyped and (0.553) for genotyped individuals.

### Prediction accuracy

In contrast, prediction accuracy estimated as the correlation between predicted genomic breeding values and estimated pedigree-based breeding values (r1) preferred using ABLUP over HBLUP, especially in populations with low to moderate heritability. Accuracies reached values from 0.729 in a population with the highest heritability to 0.830 in a population with the lowest heritability using ABLUP. The default HBLUP resulted in an increased accuracy of 0.746 in a population with the highest heritability but a decreased accuracy of 0.801 in a population with the lowest heritability. Similar results were observed in HBLUP1 scenario (Table 3). Comparing the prediction accuracy among genotyped and non-genotyped sets of individuals, a higher prediction accuracy (0.812) was evident in the non-genotyped individuals compared with the genotyped individuals (0.792) in populations with a low heritability. The opposite pattern (0.676 vs. 0.794 using default HBLUP and 0.663 vs. 0.807 using HBLUP1) was found in populations with the highest heritability (Table 3). Very similar results were evident when comparing HBLUP1 and default HBLUP, with some mixed trends. However, the implementation of any HBLUP strategy has a positive effect on the prediction accuracy of genotyped individuals in populations with higher heritability (Table 3). When alternative approach for estimation of prediction accuracy (r2) was implemented, the trends in prediction accuracy were similar to traits with high heritability while opposite to traits with low heritability. In these cases, the prediction accuracy for genotyped individuals achieved higher values compared to non-genotyped individuals. Additionally, while the prediction accuracy for non-genotyped individuals decreased compared to values obtained when estimated using alternative method (r1), higher values were reached for genotyped individuals (Table 3).

**Table 3:** Predictive ability (PA) and prediction accuracies estimated by two implemented strategies in parenthesis (r1, r2) of phenotypes for non-genotyped (NG), genotyped (G) and total (T) population using only pedigree (ABLUP), pedigree and markers using standard weighting (HBLUP) and pedigree and markers using weighting derived from the model showing best fit in terms of DIC using genotypic values (HBLUP1)

		**Kaingaroa**	**Kinleith 1**	**Kinleith 2**	**Kinleith 2**	**Kinleith 2**
**Model**	**Pop.**	**Age 2**	**Age 2**	**Age 2**	**Age 3**	**Age 4**
	**NG**	NA	NA	NA	NA	NA
**ABLUP**	**G**	NA	NA	NA	NA	NA
	**T**	0.346	0.260	0.362	0.454	0.413
		(0.809,0.616)	(0.830,0.648)	(0.745,0.788)	(0.729,0.746)	(0.738,0.795)
	**NG**	0.270	0.224	0.320	0.373	0.322
		(0.758,0.480)	(0.812,0.558)	(0.726,0.697)	(0.676,0.613)	(0.688,0.620)
**HBLUP**	**G**	0.409	0.282	0.433	0.584	0.477
		(0.804,0.728)	(0.792,0.703)	(0.759,0.942)	(0.794,0.960)	(0.743,0.918)
	**T**	0.357	0.254	0.375	0.488	0.404
		(0.790,0.635)	(0.801,0.633)	(0.738,0.816)	(0.746,0.802)	(0.718,0.778)
	**NG**	0.253	0.231	0.312	0.357	0.327
		(0.747,0.450)	(0.817,0.576)	(0.722,0.679)	(0.663,0.587)	(0.690,0.629)
**HBLUP1**	**G**	0.415	0.285	0.428	0.593	0.474
		(0.831,0.738)	(0.832,0.710)	(0.775,0.932)	(0.807,0.975)	(0.759,0.912)
	**T**	0.353	0.259	0.369	0.485	0.405
		(0.801,0.628)	(0.825,0.646)	(0.745,0.803)	(0.746,0.797)	(0.727,0.779)

The prediction accuracy of genomic prediction models between environments and ages showed similar pattern compared to predictive ability. The lowest prediction accurracy was at Kinleith 1 - age 2, with values from 0.478 to 0.534 for non-genotyped individuals and from 0.520 to 0.659 for genotyped individuals. The highest prediction accuracy was reached between Kinleith 1 - age 2 and Kaingaroa - age 2 (0.663) for non-genotyped and between Kinleith 2 - age 3 and Kinleith 2 - age 4 (0.724) for genotyped individuals (Table 4).

**Table 4:** Predictive ability and prediction accuracy in parenthesis (r1) of phenotypes for non-genotyped (NG) (above diagonal), genotyped (G) (below diagonal) across ages and environments using HBLUP1 model

**Population**		**Kaingaroa**	**Kinleith 1**	**Kinleith 2**	**Kinleith 2**	**Kinleith 2**
	**Age**	**Age 2**	**Age 2**	**Age 2**	**Age 3**	**Age 4**
**Kaingaroa**	**Age 2**	1	0.223 (0.663)	0.270 (0.512)	0.388 (0.565)	0.324 (0.525)
**Kinleith 1**	**Age 2**	0.238 (0.562)	1	0.190 (0.518)	0.199 (0.534)	0.174 (0.478)
**Kinleith 2**	**Age 2**	0.379 (0.577)	0.278 (0.659)	1	0.211 (0.447)	0.204 (0.355)
**Kinleith 2**	**Age 3**	0.492 (0. 671)	0.298 (0.575)	0.345 (0.552)	1	0.292 (0.484)
**Kinleith 2**	**Age 4**	0.354 (0.551)	0.268 (0.520)	0.304 (0.487)	0.533 (0.724)	1

## Discussion

### Factors affecting disease expression and precision of phenotype

The current progress in global climate change has resulted in the increased occurrence and severity of needle diseases in forest trees around the globe. The incidence and severity of needle disease expression depends on many environmental factors, including soil, climate and exposure and their interactions [7, 8, 35, 36]. Vuorinen and Kurkela [37] found a positive correlation between soil fertility, especially an excess of nitrogen and phosphorus, and incidence of needle cast in Scots pine (*Pinus sylvestris*). Fertilized trees contained higher levels of both elements in their needles, and had increased needle length and incidence of disease attack compared with trees under the standard treatment. Ivory [38] reported that exposure to sunlight increased the progression of the disease. Woods *et al* [39] found that a change in the local precipitation pattern during the summer season in northwestern British Columbia resulted in optimal environmental conditions for disease expression and was related to an increased severity of *Dothistroma septosporum* attacks in lodgepole pine forests.

Tree breeding is a logical response for both natural and managed populations. Quantifying the genetic effect where local, global and temporal variation affect disease expression remains complex and unreliable. Each year, for example, large variability in environmental conditions can exist and performing reliable, timely phenotyping can be difficult. Our analysis found higher levels of genetic expression of the disease at sites/years with more disease exposure, with a corresponding higher disease damage site mean and variation (standard deviation). This follows the concept that resolution of genetic differences in tree disease tolerance/resistance can depend greatly on disease incidence [40*–*42*]. This challenge in consistent phenotyping is seen in other conifer diseases (e.g. cyclaneusma needle cast [*16*]; Swiss needle cast [*43*] or red needle cast [*44]).

Vectors that distribute disease are usually influenced by temporal weather/envi- ronmental conditions, and thus the frequency of disease incidences can be spatially structured and accumulated into ’epicentres’ with unequal distribution across field experiments. Furthermore, the release of conidia from already affected trees is relatively local and spread is limited to 50 - 100 metres [45*]. Such factors further promote spatially dependent disease expression, resulting in even larger environmental heterogeneity which in turn underestimates heritability and the potential response to selection [*46*,*^47^]. The implementation of spatial analyses to remove this environmental heterogeneity resulted in improved heritability estimates and model fit when assessing the resistance of *P. radiata* to *Phytophthora pluvialis* [44] as well as *Dothistroma septosporum* [4]. Similarly, an uneven distribution of DNB disease expression in *P. radiata* was found in the current study (Figs. 1, 2 and 3), and the spatial correction of residuals resulted in improvements to genetic parameter estimates (Table 1). Therefore, spatial analyses should be considered when training genomic prediction models for resistance to needle diseases as a means to improve the quality of phenotypic information.

### Parameter tuning to improve accuracy of single-step genomic evaluation

The principle of genomic selection involves the training of a genomic prediction model with a training population for which both phenotypic and genomic information is available. Thereafter, the trained model can be used to predict phenotypes in another population where the individuals have only genomic information available [17*]. Single-step evaluation offers a means to include both genotyped and non-genotyped individuals in genomic analyses and thus benefit from the extensive phenotyping efforts that are usually part of the evaluation system of forest trees [*32*]. There are two steps which are critical for unbiased estimates of genomic breeding values when using single-step evaluations. Since the marker-based relationship matrix captures both contemporary and historical relatedness that predates the establishment of the pedigree-based base population [*18], it must be re-scaled to the base population defined by pedigree. In addition, a marker-based relationship matrix is not usually positive-semidefinite which is a requirement for analysis using mixed linear models, and sensible weighting of information derived from both pedigree and genetic markers is therefore required.

In this study, we evaluated step-wise weighting changes for the pedigree versus genetic marker data and determined the corresponding model fit in terms of DIC. We found that increased emphasis on genetic marker information was rewarded with increased heritability (Table 2). While the marker-based relationship matrix enables the capture of not only relatedness but also co-segregation and linkage disequilibrium between markers and QTLs [19*], the two latter factors can only be efficiently captured in populations with good phenotypic data and corresponding high heritability estimates. Capture of these two factors is still possible for low-heritability traits, but the size of the required training population is very large [*48*]. However, predictions based mainly on LD between markers and QTLs are still the most precise, with greater transfer-ability across generations [*49*]. Therefore, the GS prediction model re-training in future generations is required when prediction relies mostly on the current structure of relatedness in the training population [*50].

### Potential of genomics to predict host resistance

Heritability, along with the size of the training population and the effective number of chromosomal segments involved in trait expression, are all factors that contribute to the efficiency of genomic prediction [51*]. Our study found that ontogenetic stage of the studied individuals was a key factor in the precise scoring of DNB. As two-year-old individuals still have poorly developed crowns and only a few branches, especially on less fertile sites, the assessment of the proportion of the crown that is affected by diseases on the twenty one-degrees scale (from 0% to 100% with 5% steps) is challenging, even in a year with extensive disease expression. A similar pattern between tree age and heritability was observed in a previous study [*11]. However, the same study did not find any connection between the intensity of attack (stand mean infection at the individual level) and heritability. Regardless, identification of the most informative ontogenetic stage of plants is required for reliable phenotyping.

Our study found a stable pattern in resistance to DNB across ages resulting in a high prediction accuracy of 0.724 with good phenotypic expression. Similar results were found in a previous study that explored results over several field experiments, with average genetic correlations between different environments ranging from 0.72 to 0.76, depending on the genetic composition of the populations tested, and a slightly lower genetic correlation across different ages of 0.68 [11*]. In contrast, Li et al. [*52*] reported genotype by environment interaction (GxE) among environments across New Zealand using a factor analytic approach [*53*]. However, this statistical approach is confounded with connectivity so that lower genetic correlations may reflect low levels of genetic relatedness (low number of shared parents) among environments. Due to small data set, incompatible with a comprehensive approach, and previous studies of the same needle disease with extensive data, showing low GxE (when genetic connectivity between environments is sufficient) [*52], our study did not explore genotype by environment interaction.

The predictive ability in terms of correlations between genotypic values (used as phenotypes in the training process of GS prediction model) and predicted genomic breeding values showed a clear trend that reflected the heritability for each trait. In contrast, the prediction accuracy was highest for traits with the lowest heritability. This unusual pattern was likely due to the fact that the genomic breeding values for these traits were predicting the population mean rather than the deviation from the population mean caused by genetic effects. Since pedigree-based breeding values were also accumulated around the population mean (Fig. 4), the resulting correlations were relatively high. Considering both pedigree-based and predicted genomic breeding values as true breeding values and errors related to these estimates, any shared proportion of the errors would result in upward bias of correlation between them (ie. prediction accuracy) [54]. This issue was partially improved when alternative methods for estimation of prediction accuracy (r2) was implemented, especially for traits with lower heritability. On the other hand, genomic breeding values estimated for traits with high heritability were able to predict the individual deviations from the population mean caused by genetic effects, supported by both the high correlation between pedigree-based breeding values and predicted genomic breeding values (Table 3) and the observed wider dispersion of additive genetic effects around the population mean (Fig. 4). Shrinkage of breeding value estimates depends on both the phenotype errors and on the amount of information available from related individuals [55]. In our study, a low level of expression (ie. phenotype) caused either by sub-optimal weather conditions or the immature ontogenetic stage of plants to achieve reliable phenotyping likely produced large errors in the estimated breeding values and shrunk their distribution toward the population mean. This large amount of environmental variation and lack of a clear genetic signal also appeared to cause the genomic predictions to be shrunk towards the population mean. Therefore, the optimal ontogenetic stage for phenotyping and sufficient disease expression are both critical to achieve reliable genetic progress and accurate prediction in disease resistance traits.

Improvements in capturing phenotypes in the field could be enhanced through the development of more quantitative assessments using remote sensing [56*–*58*], replacing the subjective visual assessment methods presently in place. In addition, screening systems where environmental conditions are controlled could be advantageous in discovering the underlying genetic signal. The use of laboratory-based assays are under development as a possible option for discovering a stronger genetic signal, although even within more controlled conditions, variation remains a challenge [*59]. Detached needle based experiments are especially useful in situations where two diseases often co-occur in the field and genetic signals can be confounded, for example, *Phytophthora pluvialis* and *Phaeocryptopus gaeumannii* causing Swiss needle cast in Douglas-fir [60].

The lack of GxE interaction in resistance to DNB may also be explained by the low genetic diversity of *Dothistroma septosporum* possibly due to the missing sexual stage *Mycosphaerella pini* Rostr. ex Munk, identified only in Europe and Canada to date [2*,*^3^]. Analysis of New Zealand’s population of *Dothistroma* has found very limited genetic diversity, supporting conclusion that all isolates belonged to only one mating type [61*]. This single mating type has subsequently been shown to be the same type detected in Australia [*62*]. Therefore, the results of this study should be considered mating-type specific and not necessarily applicable in environments affected by other mating types. Thus, when interpreting our results, we must consider not only the context of the trajectory of climate change but also the biology and genetic diversity of the pathogen population. [*63].

## Conclusions

Implementation of genomic selection in the test population achieved a relatively high prediction accuracy compared with a pedigree-based approach. This result is promising and implies that genomics is a promising option to minimise the current biological/time constraints on reliable disease phenotyping. Reliable phenotyping, however, has to be available in the first instance, for the successful training of the prediction model. Finding the best ontogenetic stage for phenotyping plants and exposure to optimal weather conditions for disease expression, as well as the use of statistical approaches to remove environmental heterogeneity, are all important for successful prediction of resistance to needle diseases.

## Methods

### Material

The genomic prediction model was trained on three clonally replicated full-sib experiments established and managed by New Zealand Radiata Pine Breeding Company Ltd (RPBC). The Kaingaroa site (Latitude S 38^∘^ 22’ 19.5”, Longitude E 175^∘^ 55’ 41.5”) was established using an optimal design [64] comprising of single tree plot, incomplete blocks with 86 blocks each containing 36 trees with spacing of 3.1 m x 3.1 m. There were 1381 genotypes with 1–12 ramets each (mean 2.24), with the experiment containing 3096 trees in total. Similarly, Kinleith 1 (Latitude S 38^∘^ 26’ 44.0”, Longitude E 176^∘^ 30’ 22.9”) and Kinleith 2 (Latitude S 38^∘^ 18’ 52.7”, Longitude E 176^∘^ 00’ 51.2”) sites were established using the optimal design [64*] with single tree plot, incomplete blocks with 86 and 100 blocks. respectively, with 36 trees per block. There were 1270 genotypes with 1 - 9 ramets each (mean 2.02) at Kinleith 1, with a total of 3,095 trees. At Kinleith 2, there were 653 genotypes with 5 ramets each and 340 control individuals with no replicates, giving a total of 3,590 trees. The number of individuals that were genotyped was 720 at Kaingaroa, 647 at Kinleith 1, and 342 at Kinleith 2. The level of DNB expression was scored as a percentage of the crown that was affected in 5% steps [*65]. Assessments for Kaingaroa and Kinleith 1 were available for age 2, while Kinleith 2 had assessments from ages 2, 3 and 4. The expression appeared to be suppressed for age 2 assessments and data transformation via a square root function was performed.

Genomic data were generated through exome capture - genotyping-by-sequencing approach [21] using genomic resources based on resequencing of transcriptome extracted from compression wood xylem, spring xylem, summer xylem, summer phloem, spring buds, autumn buds, healthy needles, needles infected by *Phytophtora pluvialis*, seedling phloem and seedling xylem [66*]. Captured markers were removed if heterozygosity shown in megagametophyte tissues was higher than 5%, average read depth less than 10, multiallelic status, singletons and additionally each datapoint was classified as missing if ratio between reference and alternative allele was lower than 0.1 and number of read was less that 10 [*67]. The marker data were further refined for minor allele frequency (MAF) ≥0.05, and missing data were replaced by the mean genotype.

### Statistical analysis

Genotype values were used as corrected phenotypes to train the genomic prediction model, and estimated through the following mixed linear model implemented in ASReml-R statistical package [68]:

$$\boldsymbol{y}=\boldsymbol{X}\boldsymbol{\beta}+\boldsymbol{Zg}+\boldsymbol{Zb}+\boldsymbol{e} $$

where ***y*** is the vector of measurements, ***β*** is the vector of fixed effects containing the overall mean, ***g*** is the vector of random effects containing genotype effects following var(***g***) ∼N(0,***I***$\sigma _{g}^{2}$), where $\sigma _{g}^{2}$ is the genotypic variance and ***I*** is the identity matrix, ***b*** is the vector of random incomplete block effects following var(***b***) ∼N(0,***I***$\sigma _{b}^{2}$), where $\sigma _{b}^{2}$ is the incomplete block variance, ***X*** and ***Z*** are incidence matrices associating fixed and random effects to the vector of phenotypes ***y***. The residual structure was divided into spatially dependent and independent parts [46] as follows:

$$R=\sigma_{\gamma}^{2}[AR1(\rho_{col})\bigotimes AR1(\rho_{row})]+\sigma_{\delta}^{2}\boldsymbol{I} $$

where $\sigma _{\gamma }^{2}$ is the spatially dependent variance, AR1(*ρ*) is the first-order autoregressive correlation matrix, $\bigotimes $ is the Kronecker product and $\sigma _{\delta }^{2}$ is the spatially independent residual variance. Alternatively, the model was updated for including additive genetic effects to estimate narrow sense heritability, as follows:

$$\boldsymbol{y}=\boldsymbol{X}\boldsymbol{\beta}+\boldsymbol{Za}+\boldsymbol{Zg'}+\boldsymbol{Zb}+\boldsymbol{e} $$

where a is the vector of random additive genetic effects following var(***a***) ∼N(0,***A***$\sigma _{a}^{2}$), where ***A*** is the average numerator relationship matrix [69] and $\sigma _{a}^{2}$ is the additive genetic variance. In this model, ***g’*** is the vector of random non-additive genetic effects following var(***g’***) ∼N(0,***I***$\sigma _{g'}^{2}$), where $\sigma _{g'}^{2}$ is the non-additive genetic variance. All other terms in the model remained unchanged.

The narrow sense heritability was estimated as follows:

$$ \widehat{h}^{2}=\frac{\widehat{\sigma}_{a}^{2}}{\widehat{\sigma}_{a}^{2}+\widehat{\sigma}_{g'}^{2}+\widehat{\sigma}_{e}^{2}} $$

and broad-sense heritability was estimated as follows:

$$ \widehat{H}^{2}=\frac{\widehat{\sigma}_{g}^{2}}{\widehat{\sigma}_{g}^{2}+\widehat{\sigma}_{e}^{2}}. $$

The single-step genomic evaluation was performed using a mixed linear model based on a Gibbs sampling algorithm implemented in "BGLR" R package [70] as follows:

$$\boldsymbol{y}=\boldsymbol{X}\boldsymbol{\beta}+\boldsymbol{Zu}+\boldsymbol{e} $$

where ***y*** is the vector of genotypic effects estimated in the previous step, ***β*** is the overall mean assigned with a flat prior, ***u*** is the vector of genomic estimated breeding values following var(***u***) ∼MVN(0,***H***$\sigma _{u}^{2}$), where $\sigma _{u}^{2}$ is the marker-based additive genetic variance with prior density following the default setting of ∼*χ*^−2^(df=5, S=var(***y***)*0.5), and ***H*** is the relationship matrix, incorporating information from both the pedigree and genomic markers, and is constructed as follows:

$$\boldsymbol{H}=\left[\begin{array}{cc} \boldsymbol{A_{11}}+\boldsymbol{A_{12}A_{22}^{-1}}(\boldsymbol{G_{w}}-\boldsymbol{A_{22}})\boldsymbol{A_{22}^{-1}A_{21}} & \boldsymbol{A_{12}A_{22}^{-1}G_{w}}\\ \boldsymbol{G_{w}A_{22}^{-1}A_{21}} & \boldsymbol{G_{w}}\\ \end{array}\right] $$

where ***A***_***11***_ is the pedigree-based relationship matrix for non-genotyped individuals, ***A***_***22***_ is the pedigree-based matrix for genotyped individuals, ***A***_***12***_ and ***A***_***21***_ are the pedigree-based matrices between genotyped and non-genotyped individuals, ***G***_***w***_ is the rescaled and weighted marker-based relationship matrix. The marker-based relationship matrix was estimated according to [71] as follows:

$$\boldsymbol{G}=\frac{\boldsymbol{ZZ}'}{2\sum_{j}p_{j}(1-p_{j})} $$

where ***Z*** = ***M*** - ***P***, ***M*** is the genotype matrix with the reference allele homozygote coded as 0, a heterozygote as 1 and the alternative allele homozygote as 2 (reference and alternative alleles are defined relative to the *Pinus taeda* reference genome v. 1.01e [72]) and ***P*** is double the frequency for the alternative allele. Since the marker-based relationship matrix is reflecting both contemporary relatedness, as defined by the documented pedigree, and historical relatedness, that existed prior to the development of the base population as defined by the pedigree [18*,*^73^*], therefore on a different scale to the pedigree-based relationship matrix. Therefore, rescaling of the marker-based relationship matrix was required. We adopted the rescaling approach developed in Gao et al. [*74] as follows:

$$\left\{\begin{array}{l} Avg.diag(\boldsymbol{G})\beta + \alpha = Avg.diag(\boldsymbol{A_{22}})\\ Avg.offdiag(\boldsymbol{G})\beta + \alpha = Avg.offdiag(\boldsymbol{A_{22}}) \end{array}\right. $$

The marker-based relationship matrix is often not positive semi-definite, which is one of the requirements of mixed linear models for covariance structures, and thus the weighting of information derived from genomic markers and pedigree has to be performed. We defined a weighting of 0.05 for pedigree information as our default scenario (HBLUP), however, all other weighting scenarios were tested at 0.1 increments to determine the optimal weighting for each separate trait (HBLUP1). Weighting scenarios were evaluated on the basis of Deviance Information Criterion (DIC) which is equivalent to Akaike’s Information Criterion (AIC) in the Bayesian framework (lower value represents better model fit). The weighting of marker-based and pedigree-based relationship matrices was performed as follows:

$$\boldsymbol{G}_{w}=\boldsymbol{G}(1-w)+\boldsymbol{A}_{22}w $$

where w is the proposed weighting for the pedigree-based relationship matrix. Similar to the vector ***u***, ***e*** is the vector of residual effects following var(***e***) ∼MVN(0,***I***$\sigma _{e}^{2}$), where ***I*** is the identity matrix and $\sigma _{e}^{2}$ is the residual variance with a prior density following the default setting ∼*χ*^−2^(df=5, S=var(***y***)*0.5). The number of iterations was set to 120,000, burnIn to 20,000, and thinning set to 10. Additionally, the same mixed linear model was performed using pedigree-based relationship matrix [69] instead of ***H*** matrix to investigate pedigree-based scenario (ABLUP).

Independent evaluation of the prediction model was performed through 10-fold cross-validation, where one tenth of individuals were iteratively defined as the validation population, and all the phenotypes from these individuals were masked as missing values. Predicted values from these individuals were then correlated with both genotypic values to determine predictive ability (PA) and with pedigree-based estimated breeding values to determine prediction accuracy (r1). Alternatively, the prediction accuracy (r2) was estimated as follows:

$$r2=\frac{cor(GEBV,y)}{\sqrt{h^{2}}} $$

where the nominator is the predictive ability (correlation between predicted genomic breeding values and corrected phenotypes) and the denominator is the square root of the heritability [75]. The heritability used in the estimate of the prediction accuracy (r2) was inferred from the model using a spatial analysis due to better model fit.

## Data Availability

Data used in the analysis are publicly available from DRYAD data repository doi:10.5061/dryad.qfttdz0d7.

## Abbreviations

DNB: Dothistroma needle blight
GS: Genomic selection
QTL: Quantitative trait loci
DIC: Deviance information criterion
ABLUP: Pedigree-based mixed linear model
HBLUP: Single-step genomic evaluation using default weighting of pedigree and marker information
HBLUP1: Single-step genomic evaluation using weighting of pedigree and marker information that gives the best model fit (the lowest DIC score)
LD: Linkage disequilibrium
GxE: Genotype by environment interaction
RPBC: Radiata Pine Breeding Company Ltd.
MAF: Minor allele frequency
AIC: Akaike’s information criterion
